# Remote Activation of H–H Bonds by Platinum in Dilute Alloy Catalysts

**DOI:** 10.1021/acscatal.4c00886

**Published:** 2024-04-23

**Authors:** Tongxin Han, Yuanyuan Li, Tao Wu, Debora Motta Meira, Shuting Xiang, Yueqiang Cao, Ilkeun Lee, Xing-Gui Zhou, De-en Jiang, Anatoly I. Frenkel, Francisco Zaera

**Affiliations:** † Department of Chemistry and UCR Center for Catalysis, University of California, Riverside, Riverside, California 92521, United States; ‡ Department of Materials Science and Chemical Engineering, Stony Brook University, Stony Brook, New York 11794, United States; § The State Key Laboratory of Fine Chemicals, School of Chemical Engineering, Dalian University of Technology, Dalian 116024, P. R. China; ∥ CLS@APS, Advanced Photon Source, Argonne National Laboratory, Argonne, Illinois 60439, United States; ⊥ Canadian Light Source Inc., 44 Innovation Boulevard, Saskatoon, Saskatchewan S7N 2V3, Canada; # State Key Laboratory of Chemical Engineering, School of Chemical Engineering, East China University of Science and Technology, Shanghai 200237, P. R. China; ∇ Department of Chemical and Biomolecular Engineering, Vanderbilt University, Nashville, Tennessee 37212, United States; ○ Chemistry Division, Brookhaven National Laboratory, Upton, New York 11973, United States

**Keywords:** single-atom alloy (SAA) catalysts, *in situ* studies, X-ray absorption spectroscopy (XAS, XANES, EXAFS), density functional theory (DFT), infrared absorption spectroscopy (IR), segregation, diffusion, hydrogenation catalysis

## Abstract

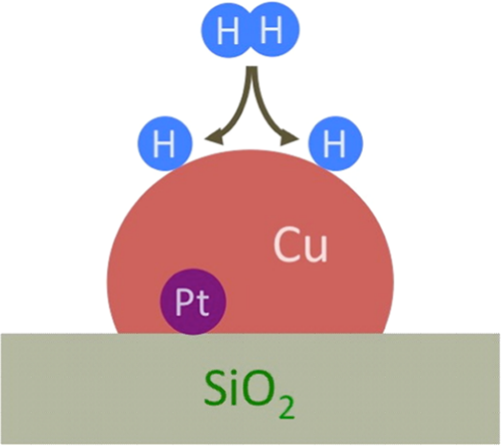

With heterogeneous catalysts, chemical promotion takes place at their surfaces. Even in the case of single-atom alloys, where small quantities of a reactive metal are dispersed within the main host, it is assumed that both elements are exposed and available to bond with the reactants. Here, we show, on the basis of *in situ* X-ray absorption spectroscopy data, that in alloy catalysts made from Pt highly diluted in Cu the Pt atoms are located at the inner interface between the metal nanoparticles and the silica support instead. Kinetic experiments indicated that these catalysts still display better selectivity for the hydrogenation of unsaturated aldehydes to unsaturated alcohols than the pure metals. Density functional theory calculations corroborated the stability of Pt at the metal–support interface and explained the catalytic performance as being due to a remote lowering of the activation barrier for the dissociation of H_2_ at Cu sites by the internal Pt atoms.

## 1 Introduction

Much heterogeneous catalysis relies on the promotion of the dissociation of diatomic molecules by metals. The hydrogenation of organic reactants, for instance, typically requires an initial H–H bond-scission step, a reaction easily facilitated by platinum-group transition metals.^1^ Unfortunately, platinum metals do not discriminate well among the various possible hydrogenation steps that the organic reactants follow afterward, and therefore offer poor selectivity when the aim is to synthesize a particular product. One way to address this limitation that has gained much attention in recent years is to dilute a small amount of the platinum-group atoms within a second less active but more selective element, typically a coinage metal, in order to combine the H_2_ activation ability of the dopant with the milder chemistry of the host.^2^

The effectiveness of this so-called single-atom alloy (SAA) approach has been empirically proven for a number of reactions.^3,4^ In particular, we have recently demonstrated the gain in selectivity induced by the added Pt in 5 wt % CuPt_*x*_/SBA-15 catalysts consisting of Cu nanoparticles (NPs) doped with an x molar fraction of Pt and dispersed on SBA-15 (a mesoporous silica support) for the hydrogenation of unsaturated aldehydes.^5,6^ The way that these SAA catalysts operate, however, is still under discussion. Much elegant surface-science work using model metal surfaces such as single crystals and controlled ultrahigh vacuum (UHV) environments has been performed^4,7^ and complemented with quantum mechanics calculations^8^ to support a proposed mechanism by which H_2_ molecules are dissociated on individual Pt or Pd atoms, which are allegedly present on the surface, after which the resulting adsorbed H atoms spill over to the Cu or Au surfaces where the organic molecules are hydrogenated. In the case of the hydrogenation of unsaturated aldehydes, a combination of infrared absorption spectroscopy (IR) and temperature-programmed desorption (TPD) experiments, together with complementary quantum mechanics calculations, has shown that, indeed, Cu surfaces provide the hydrogenation selectivity lacking with Pt, a difference justified on the basis of the differences in coordination geometry of the reactants to the two metals.^9,10^

When considering realistic catalytic conditions, namely, when using catalysts consisting of SAA NPs dispersed on a high-surface-area oxide support and atmospheric pressures or liquid solutions, additional factors come into play, and that brings into question some of the features of the model mentioned above. Specifically, evidence from our group and from others has indicated that the performance of real SAA catalysts may be affected by the ease with which metals segregate to the surface and/or diffuse into the metal bulk upon exposure to various chemical environments.^5,11−13^ Here, we have combined results from the *in situ* characterization of Cu–Pt SAAs under hydrogen and carbon monoxide atmospheres with quantum mechanics calculations to explore this phenomenon directly. We find that, indeed, the Pt atoms diffuse away from the surface under typical hydrogenation conditions but still assist remotely with the dissociation of molecular hydrogen. The details are provided below.

## 2 Methods and Protocols

The CuPt_*x*_/SBA-15 catalysts were prepared using an incipient-wetness impregnation method.^5^ Defined amounts of copper nitrate (Cu(NO_3_)_2_·3H_2_O, Sigma-Aldrich, 98%) and chloroplatinic acid (H_2_PtCl_6_·6H_2_O, Sigma-Aldrich, ≥37.50% Pt basis) were mixed with deionized water to obtain the desired metal loadings, 5 wt % Cu and 0.076 wt % Pt for the CuPt_0.005_/SBA-15 discussed here. About 2 g of commercial SBA-15 (ACS Material), a silica mesoporous material with well-defined one-dimensional pores of approximately 7 nm in diameter, was impregnated with the liquid mixture, kept at room temperature for 24 h, dried at about 355 K for 24 h, and ground to a powder. Before use, approximately 0.1 g of the catalyst was loaded in a furnace tube and pretreated by heating it to 625 K under Ar (flow rate, 40 mL/min), reducing it at 625 K under H_2_ (flow rate, 50 mL/min) for 3 h, and cooling it down to room temperature under Ar (flow rate, 40 mL/min). X-ray photoelectron spectroscopy (XPS) was used to confirm the full reduction of the metals.^5^

**Figure 1:**
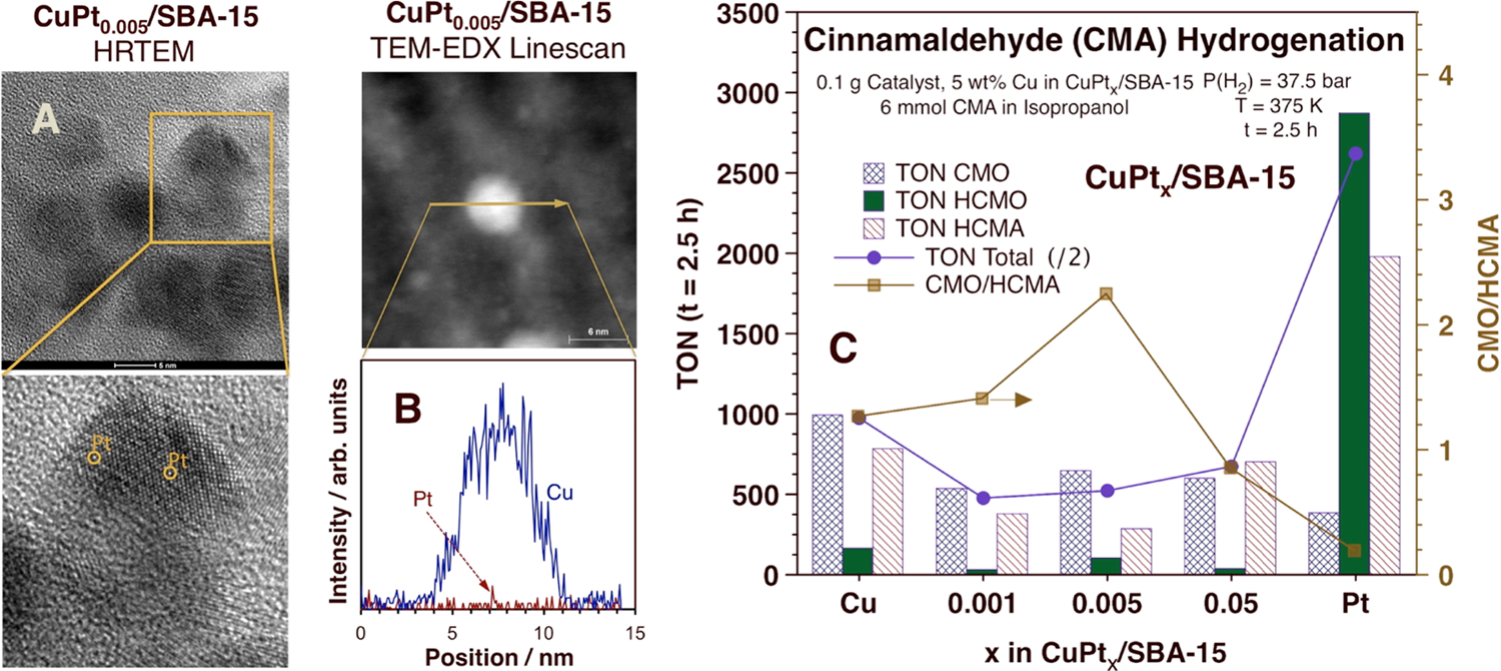
CuPt*_x_*/SBA-15 catalyst characterization and performance. (A) Dark-field HRTEM images of CuPt_0.005_/SBA-15 highlighting the dispersed nature of the Pt atoms. The edges of the yellow square, a zoomed-in view of which is shown in the bottom panel, are approximately 15 nm in size. (B) Bright-field Cu (blue) and Pt (red) TEM-EDX line scans across an individual CuPt_0.005_ NP. (C) Typical catalytic performance of CuPt_*x*_/SBA-15 catalysts for the hydrogenation of cinnamaldehyde (CMA) in terms of turnover numbers (TONs) for the total conversion of the reactant (purple filled circles) and for all three products (cinnamyl alcohol −CMO–, crosshatched blue bars; 1,2-dihydrocinnamyl alcohol −HCMO–, solid green bars; and 3-phenylpropanal −HCMA– hatched red bars) after 2.5 h of reaction (left scale) and of the ratio of CMO to HCMA (right scale, gold filled squares), all as a function of the molar fraction of Pt (*x*) in the dilute alloy NPs.

Metal loadings were quantified by inductively coupled plasma atomic emission spectrometry (ICP-AES), using a PerkinElmer Optima 7300DV ICP-OES apparatus.^6^ The actual metal loadings in the CuPt_0.005_/SBA-15 sample determined this way were 4.8 wt % Cu and 0.073 wt % Pt. The metal NPs’ size distribution was estimated to be approximately ⟨*d*⟩ = 6 ± 2 nm by scanning transmission electron microscopy (STEM), using a FEI Titan Themis 300 STEM instrument; a Bruker Quantax attachment was used to carry out energy-dispersive X-ray spectroscopy (EDX) imaging to obtain the NP chemical composition profiles shown in Figures 1B and S1. The high-resolution transmission microscopy (HRTEM) images shown in Figure 1A and S2 were acquired with the same FEI Titan Themis 300 instrument.

Catalytic performances were tested using a 300 mL high-pressure 4560 bench Parr batch reactor.^5,6^ The reaction mixture was prepared in the reaction vessel by mixing 75 mL of isopropanol (Sigma-Aldrich, ≥99.7% purity, used as the solvent), 0.8 g of cinnamaldehyde (CMA, Sigma-Aldrich, ≥95% purity), and 2 mL of benzyl alcohol (Sigma-Aldrich, 99.8% purity, used as an internal standard) and then adding approximately 0.1 g of the catalyst. The vessel was purged five times with 10 bar of H_2_ (Liquid Carbonic, >99.995% purity) and then pressurized to the desired reaction H_2_ pressure, 37.5 bar (at 375 K) in the experiments reported in Figure 1C. After heating to the reaction temperature (375 K), stirring was switched on, and the time was set to 0. 1 mL aliquots were taken at preset times (2.5 h in Figure 1C) and analyzed using an Agilent 6890N gas chromatograph with an HP-50 column (15 m × 320 μm × 0.25 μm) to determine their composition. Turnover numbers (TONs) were estimated in terms of molecules per surface metal atom (Cu or Pt) by using the NP size distribution measured by TEM and assuming spherical shapes and bulk metal densities.

The *in situ* X-ray absorption spectroscopy (XAS) experiments were carried out at the 20-ID-B,C beamline of the Advanced Photon Source (APS) of Argonne National Laboratory. A Si(311) double-crystal monochromator and a toroidal focusing mirror were employed. The third harmonic of the undulator was used, with full scanning. The beam was 500 × 800 μm^2^ in size. The Pt L_3_-edge and Cu K-edge data were collected in fluorescence mode using a four-element Vortex detector with a cube preamplifier operated at about 200,000 counts/s per element. Eight layers of aluminum were added to suppress the Cu fluorescence background. A capillary furnace composed of a resistively heated quartz tube (O.D. = 1.5 mm, I.D. = 1.3 mm) was loaded with the catalyst in powder form. Initial spectra were taken at room temperature in flowing He (pure, 10 mL/min), after which the catalyst was reduced at 625 K for 3 h with a 5 vol % H_2_ in N_2_ gas mixture (flowing rate = 10 mL/min) and cooled down to room temperature in the same atmosphere; new sets of XAS spectra were then taken. This treatment was shown to be sufficient to fully reduce the metals to their zerovalent states; the Cu K-edge XAS data corroborating this fact are provided in Figure S3. The temperature was subsequently cycled several times between 295 K (room temperature) and 495 K, and spectra were recorded *in situ* with the catalyst continuously exposed to the 5 vol % H_2_/N_2_ gas stream. Similar *in situ* temperature cycling experiments were performed using pure He and 5 vol % CO in He gas streams (all at flowing rates of 10 mL/min).

XAS spectra were processed and analyzed using the Demeter package.^14^ For the extended X-ray absorption fine structure (EXAFS) data analysis, the *k* and *R* fitting ranges were 3–12.5 Å^–1^ and 1.5–3.2 Å, respectively. The amplitude reduction factor (0.84 ± 0.04) was estimated by fitting the data obtained for a reference Pt foil. In fitting the spectra for CuPt_0.005_/SBA-15, a model that included Pt–O, Pt–Cu, and Pt–Pt paths was tested first. However, a large shift in the threshold energy (Δ*E*_0_) was obtained (>20 eV) for the Pt–O path, suggesting that the peak at about 1.7 Å in the R-space EXAFS spectra was most likely not due to a Pt–O contribution. Subsequently, a model that included paths for Pt–Si, Pt–Cu, and Pt–Pt coordinations was chosen; this approach yielded by far the best results, as summarized in Figure S4 and Table S1. To further support our choice of path fitting, additional experiments were carried out using a second catalyst with a higher Pt content (CuPt_0.2_/SBA-15) oxidized in air. This way, it was determined that the peak for Pt coordinated to oxygen atoms is expected at approximately 0.1 Å lower distance than that fitted with a Pt–Si path (Figure S4a). We are confident that there is enough of a difference between the results of fitting the data with Pt–O vs Pt–Si paths to conclude that in our catalyst some of the Pt atoms are indeed coordinated to Si, not O, atoms. The X-ray absorption near-edge structure (XANES) portion of the XAS spectra was simulated using the FEFF9 code.^15^ The experimental XANES of a Pt foil, a reference sample, was chosen as the standard to optimize the FEFF parameters, and the *lfms1* in the *SCF* card was changed from “0” (the value for calculations with solids) to “1” in order to perform our molecular calculations.

Density functional theory (DFT) calculations were carried out employing the Vienna ab initio simulation package (VASP).^16^ The interactions between electrons and ions were described using the projector augmented wave (PAW) method.^17^ The exchange-correlation energy was calculated using the Perdue–Burke–Ernzerhof (PBE) version of the generalized gradient approximation (GGA).^18^ The optimization of the lattice constants and atomic positions was performed with a cutoff energy of 500 eV for the plane-wave basis. Convergence criteria were set at total energies below 10^–4^ eV per atom and Hellmann–Feynman forces smaller than 0.05 eV Å^–1^. Sampling of the Brillouin zone was achieved using only one γ point due to the large unit cell used. In the process of constructing the models used in this study, a substrate comprising two layers of SiO_2_ based on the (111) surface of the β-cristobalite lattice^19^ with the Cu cluster located on the SiO_2_ surface was used. The bottom layer of the SiO_2_ slab was held at a fixed position, and the terminal oxygen atoms on the lower layer were saturated via the addition of hydrogen atoms. All other atoms were allowed to undergo relaxation. The climbing-image nudged elastic band (CI-NEB) method^20^ was used to search for the transition states of H_2_ dissociation on the catalyst surfaces.

*In situ* infrared absorption spectra (IR) for CO adsorption on the CuPt_*x*_/SBA-15 catalysts were obtained using a homemade quartz cell and a Bruker Tensor 27 Fourier-transform IR (FTIR) spectrometer equipped with a deuterated triglycine sulfate (DTGS) detector.^21^ A wafer of the catalyst was placed in the center of the transmission IR cell and reduced at 625 K under 500 Torr H_2_ for 3 h. For the experiments carried out under vacuum, the cell was evacuated and cooled down to 125 K, after which the sample was exposed to 50 Torr of CO (Matheson Tri-Gas, ≥99.5% purity) for 0.5 h and the cell was then evacuated for 10 min. Spectra were recorded from 125 to 475 at 20 K intervals as the sample and cell were warmed up and corrected using background traces obtained under the same condition before adsorption. For the *in situ* CO IR experiments, the cell was kept at room temperature: spectra were acquired at the indicated CO gas pressures and referred to corresponding background spectra taken under the same conditions but without the catalyst. All spectra were acquired with a resolution of 2 cm^–1^ and correspond to averages of 16 scans.

## 3 Results and Discussion

Figure 1A,B reports a typical high-resolution transmission electron microscopy (HRTEM) image and the energy-dispersive X-ray spectroscopy (TEM-EDX) analysis of an individual NP in our CuPt_0.005_/SBA-15 catalyst, respectively, showing the atomic dispersion of the Pt atoms within the Cu matrix in the initial catalyst, before use (additional TEM-EDX line scans and HRTEM images are provided in Figures S1 and S2, respectively).

Figure 1C illustrates the trends seen in terms of the hydrogenation activity and selectivity as a function of alloy composition. The Pt-only catalyst is quite active but mainly leads to the full hydrogenation of the reactant, cinnamaldehyde (CMA), to 1,2-dihydrocinnamyl alcohol (HCMO), and to the partial hydrogenation of the C=C double bond to produce 3-phenylpropanal (HCMA); the yield of the desired product, cinnamyl alcohol (CMO, from hydrogenation of the C=O carbonyl group), amounts to less than 15% of the total CMA conversion. The Cu-only catalyst displays better selectivity toward CMO but about an order of magnitude less activity. The addition of small amounts of Pt to the Cu NPs clearly improves the overall performance of the two monometallic catalysts: with CuPt_0.005_/SBA-15, for instance, the CMO/HCMA yield ratio increases by almost a factor of 2 relative to that of the pure-Cu case (gold filled squares in Figure 1C). The overall activity of the dilute alloy catalysts is lower than that of pure Cu, but that is mainly due to the suppression of the production of the undesirable HCMA.

For SAA catalysts to work, two main requirements have been identified in the literature: (1) the main metal, Cu in this case, must promote the hydrogenation steps selectively; (2) the dopant, Pt, must promote the activation of hydrogen but not interfere with the rest of the surface reactions. For the case of the hydrogenation of unsaturated aldehydes, we have in the past provided both experimental and theoretical evidence for the first requisite.^5,10^ The way Pt improves the performance of these catalysts, however, is not fully understood yet. Extensive surface-science experiments and density functional theory (DFT) calculations have shown that platinum–metal atoms diluted in coinage-metal matrices can indeed activate H_2_.^2,4,22^ However, that research has assumed that the atoms of the dopant are present on the surface of the alloy, and that may not be the case when dealing with realistic catalytic conditions, with dilute alloy NPs dispersed on high-surface-area supports and exposed to atmospheric pressures of reactive gases.^23^ We have also provided kinetic evidence indicating that the addition of Pt to Cu, even in dilute form, affects both the equilibrium constants for the adsorption of the reactants and products (because of changes in the nature of the surface) and the rates of most unsaturated aldehyde hydrogenation steps.^6^ Here, we introduce new *in situ* spectroscopic evidence and DFT calculations to show that during catalysis, the Pt atoms are in fact preferentially located at the NP/silica interface, away from the exposed surface of the metal NPs. Additional calculations explain how those atoms can still help activate H_2_ remotely at the surface of the NPs by disturbing the electronic structure of the Cu atoms in the alloy across several crystal lattice units and by decreasing the H–H bond-scission activation energy on the Cu surface sites.

**Figure 2:**
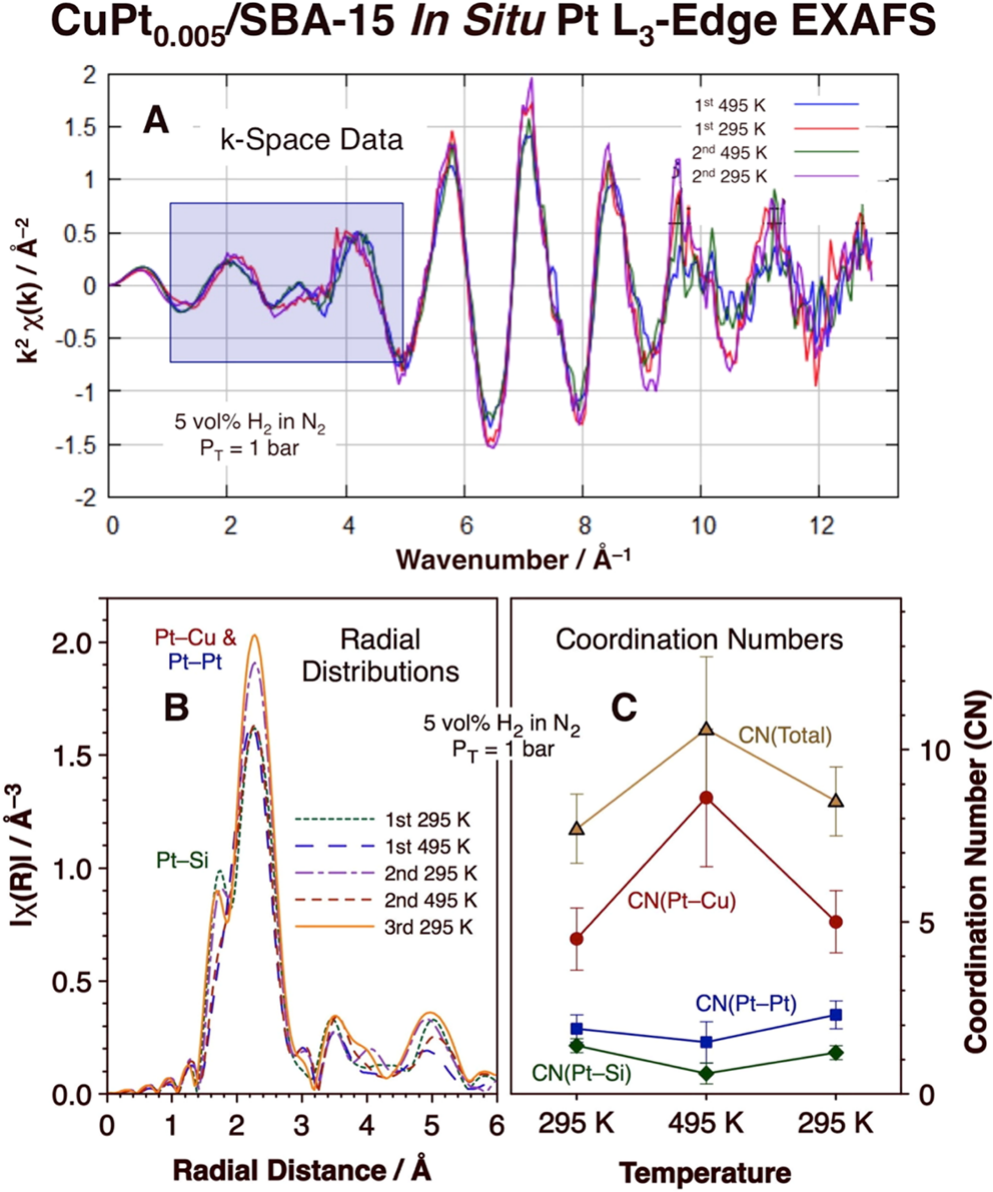
*In situ* EXAFS data for CuPt_0.005_/SBA-15 under a hydrogen atmosphere. (A) *k*-space data obtained during two cycles of heating and cooling of the catalyst between 295 and 495 K (the blue box highlights one of the regions where the most noticeable changes take place as the temperature is cycled). (B) Corresponding radial distribution data for three heating–cooling cycles. (C) Average coordination numbers obtained from analysis of the data in panel (B) as a function of temperature.

The main evidence for our claim that in the presence of H_2_ the Pt atoms reside at the inner NP/silica interface comes from X-ray absorption spectroscopy (XAS) data acquired *in situ* under such a reactive atmosphere. The key results for the Pt L_3_-edge EXAFS component of the data are reported in Figure 2. In these experiments, the CuPt_0.005_/SBA-15 catalyst was repeatedly heated to 495 K and cooled back down to 295 K under a flowing stream of N_2_ gas mixed with 5 vol % of H_2_ at a total pressure of 1 bar. The first clear observations deriving from the data are that the structure of the catalyst is different at 295 K vs at 495 K and that the changes are reversible. This can be observed both in the original χ(*k*) vs wavenumber data (Figure 2A, especially in the 1–5 and 9–12 Å^–1^ regions) and in the radial distributions obtained by Fourier transformation of the k-space data (Figure 2B). Particularly interesting is the additional peak seen in Figure 2B around 1.7 Å when the catalyst is exposed to H_2_ at room temperature, which is most likely associated with a scattering path between the Pt and Si atoms from the silica support (details about the fitting and the resulting best-fit values for coordination numbers and bond distances are provided in Figure S4 and Table S1, as already discussed in Section 2). Figure 2C summarizes the average coordination numbers around the Pt atoms calculated by our best-fit modeling of the Pt L_3_-edge EXAFS data. The average number of Si atoms coordinated to individual Pt atoms at room temperature was estimated to be 1.3 ± 0.2, a number that is reduced to 0.6 ± 0.3 upon heating to 495 K, and the total coordination number around the Pt atoms was also found to be significantly lower at 295 K vs 495 K: 8.1 ± 0.9 vs 10.6 ± 2.1. Both of these results, together with the observed limited but nonzero coordination of Pt with other Pt atoms also extracted from the EXAFS data (discussed in more detail later), indicate that at room temperature most if not all of the Pt atoms are located at the NP/silica interface.

**Figure 3:**
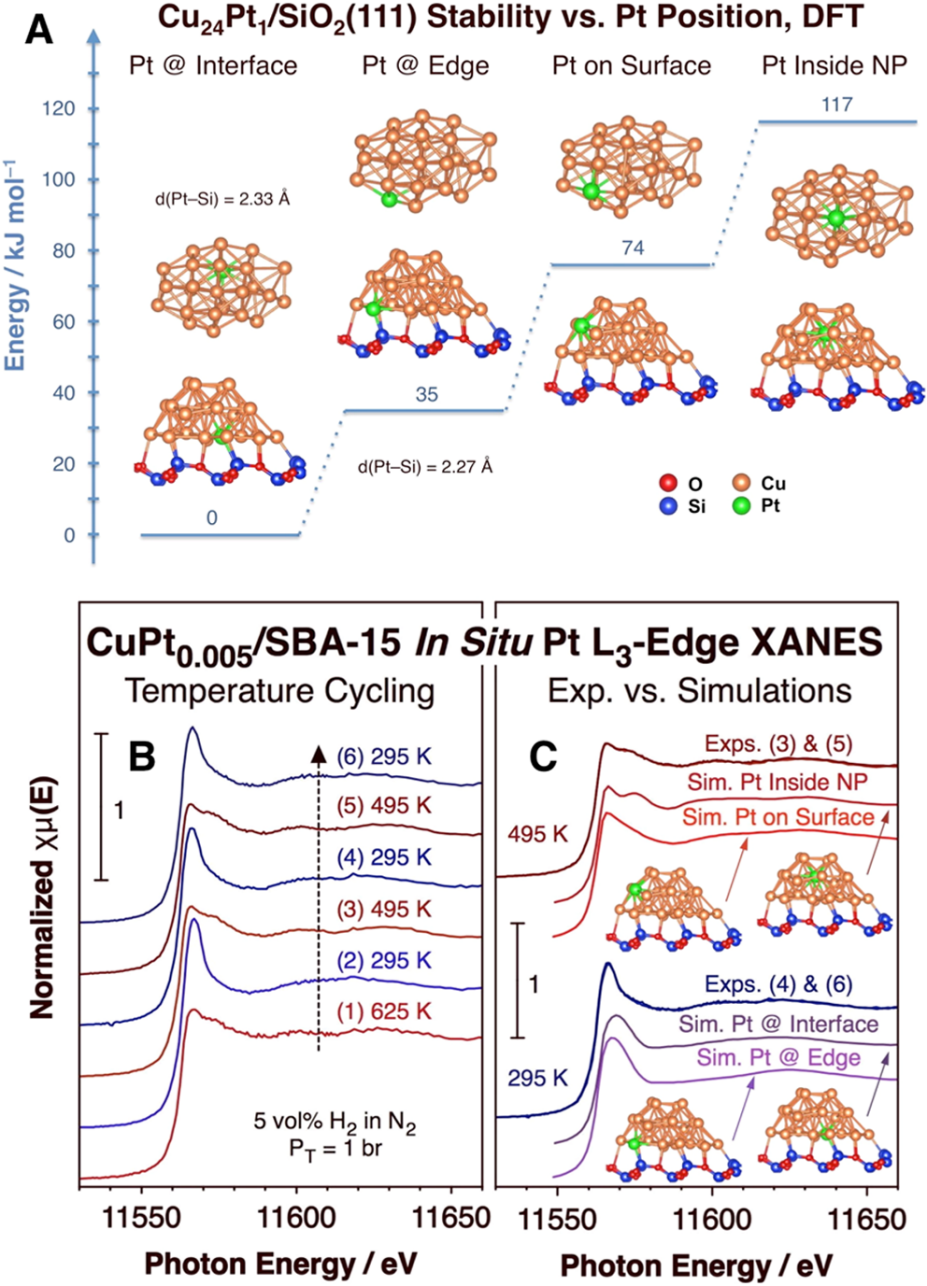
Evidence for the location of the Pt atoms in CuPt_0.005_/SBA-15 at the NP/silica interface. (A) DFT calculations of the energy of formation of Cu_24_Pt clusters on a SiO_2_(111) surface vs Pt position, relative to the case of Pt at the NP/silica interface. (B) *In situ* XANES data obtained during three heating–cooling cycles between 295 and 495 K under a 5 vol % H_2_/N_2_ atmosphere. (C) Comparison of the XANES data obtained at 295 (bottom) and 495 (top) K with simulated spectra using the cluster structures estimated by DFT in panel (A).

The fact that the Pt atoms prefer to sit at the CuPt_0.005_/SiO_2_ interface may seem surprising, but is corroborated by DFT calculations with Cu_25_ and Cu_24_Pt clusters supported on a SiO_2_(111) surface (Figure 3A). Comparison with the energetics of the formation of the free metal clusters, not bound to any support, helps us understand this result in terms of the added stability provided by the formation of a new Si–Pt bond (Figure S5). *In situ* XANES spectra are also in agreement with the EXAFS and DFT results, in particular with the reversibility of the changes as the temperature is cycled between 295 and 495 K (Figure 3B). By contrasting simulated XANES spectra for the different DFT models with the experimental XANES spectra at 295 and 495 K, it was possible to corroborate that Pt is likely to be at the inner NP/silica interface sites (or perhaps the edge) at 295 K and inside the Cu cluster (or at the surface) at 495 K (Figure 3C).

**Figure 4:**
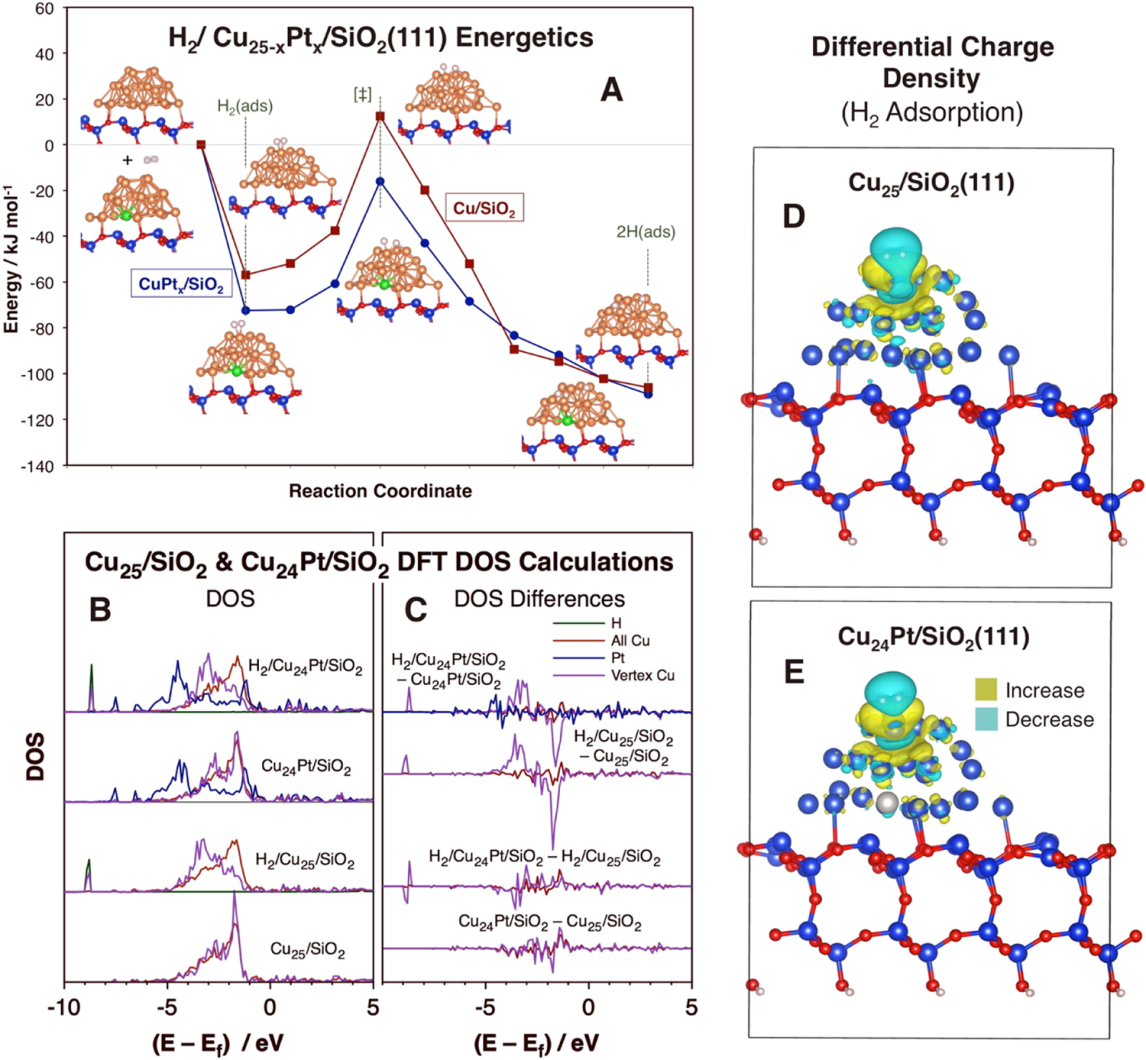
Changes in the energetics and electronic properties of CuPt*_x_*/SiO_2_(111) upon H_2_ adsorption. (A) DFT calculations of the PES along the reaction coordinate for H_2_ activation on Cu_25_ (red filled squares) and Cu_24_Pt (blue filled circles) clusters supported on a SiO_2_(111) surface. (B) Calculated DOS for both systems before and after H_2_ adsorption (normalized per atom). (C) Differential DOS upon Pt substitution in the Cu cluster (bottom two data sets) and upon H_2_ adsorption (top two sets). (D, E) Differential charge density upon H_2_ adsorption on the Cu_25_/SiO_2_(111) (D) and Cu_24_Pt/SiO_2_(111) (E) clusters. Atom color code: O = red and Si = blue in silica; Cu = blue, Pt = large gray, H = small gray in the meal cluster.

If in the CuPt_0.005_/SBA-15 dilute alloy catalyst the Pt atoms are at the inner NP/silica interface while being exposed to H_2_, how can they still exert an effect on the catalytic behavior? The answer, according to our DFT calculations, is that the Pt atoms can remotely modify the electronic properties of the surface Cu atoms and affect the energetics of the H_2_ activation. Figure 4A shows the calculated potential energy surfaces (PESs) along the reaction coordinate for both pure Cu_25_ and Cu_24_Pt SAA clusters. It is seen there that the substitution of one Cu atom with Pt at the NP/silica interface lowers the adsorption energy for H_2_ by close to 20 kJ/mol and, more importantly, also lowers the activation barrier for the dissociation of molecular H_2_ that leads to the formation of two adsorbed H atoms. The effect is large enough to bring down the energy of the activated complex below that of H_2_ molecular desorption, reversing the order that explains why Cu is not good for the promotion of hydrogenation reactions.^24^ Curiously, the Pt substitution does not appear to significantly affect the adsorption energy of atomic hydrogen.

The Pt atom is able to promote the activation of molecular hydrogen remotely because it affects the electronic structure of the Cu atoms at the surface of the cluster, even though it is several crystal lattice units away (two in the clusters used in our DFT calculations). This effect can be easily observed by the changes in the density of states (DOS) reported in Figure 4B (and in differential mode in Figure 4C). A couple of observations are worth highlighting from those results. First, the Pt substitution in the Cu_25_/SiO_2_(111) cluster at an inner NP/silica interface site induces a redistribution of the DOS in all of the Cu atoms (red traces), including in the surface Cu atom on which H_2_ adsorbs (vertex Cu; purple traces): there is a slight shift in the DOS between −1 and −2 eV toward the Fermi level, and additional electronic density develops around −3 eV at the vertex Cu. These changes contradict the general assumption that the *d* orbitals of the dopant metal in SAAs do not mix with those of the host metal and that therefore the dopant behaves as an isolated atom.^25^ A possible explanation for this discrepancy is that most past experiments and DFT calculations leading to the “isolated atom” model have involved bulk alloys, and Cu–Pt SAA clusters display DOS distributions noticeably different from those (Figure S6). We suggest that the idea of the dopant atoms in SAA catalysts acting as isolated atoms may need to be reevaluated.

Another observation from the data in Figure 4 is the fact that the adsorption of H_2_ mainly affects the vertex Cu, as expected (much of its DOS at −2 eV shifts to a range between −3 and −4 eV), but also the Pt atom: specifically, the Pt DOS shows an increase between −4 and −5 eV: in other words, not only can the Pt atom exert a change in the electronic properties of the Cu surface atoms, but also chemical changes at the surface can alter the electronic characteristics of the embedded Pt atom. The effect of H_2_ adsorption on the pure and Pt-substituted clusters can be seen in the charge densities plotted in Figure 4D,4E as well, although the changes there are subtle and more difficult to interpret. It seems that the Pt substitution in the Cu_25_/SiO_2_(111) cluster leads to a suppression of the lowering in the charge density at the sides of the H_2_ molecule upon adsorption. There is also a decrease in charge density in the Pt atom on the side facing the SiO_2_ surface.

Overall, these DFT calculations confirm in a qualitative way the experimental observation that Pt atoms prefer to migrate to the NP/silica interface at low temperatures and under H_2_, and they provide a rationale for how these embedded Pt atoms are still able to affect catalytic behavior. Nevertheless, it should be indicated that they do exhibit some limitations. The main difficulty is that the clusters used in our modeling contain only 25 atoms and form NPs approximately 1 nm in diameter; the catalysts used in the experiments are much larger NPs, approximately 6 nm in diameter. This not only means that the Pt content in the model is higher than that in the real catalyst, but also brings about questions regarding the ability of DFT calculations to truly simulate the geometric and electronic properties of the catalyst NPs realistically. Unfortunately, the state-of-the-art DFT programs are not yet capable of handling large NPs, of the dimensions used in the catalysts here. Nevertheless, we believe that the qualitative lessons derived from our calculations are robust. First, although the clusters in our model have somewhat distorted geometries, they do closely resemble the fcc structure of bulk Cu as seen in the HRTEM images of our catalysts (Figures 1 and S2). Their electronic properties are also different from those of bulk Cu (Figure S6), but they still show metallic behavior; the DOS calculated here may perhaps represent an extreme case of how the DOS varies when transitioning from the bulk metal to the NPs. We also like to point out that in our calculations with larger (84 atoms, >2 nm) NPs, as in Figure S10 (discussed later), the most stable configurations are still those with the Pt atoms at the NP/silica interface. The question remains: what is the distance over which the Pt atoms can exert their catalytic modification ability? We may not be able to answer that here, but our calculations do indicate that the effect travels for at least two, and perhaps three, lattice distances. It may be that at least some Pt atoms in the real catalysts sit at the NP/silica interface but only a few lattices away from the edge.

The Pt atoms in Cu–Pt dilute alloys may prefer to be at the inner interface between the metal NPs and the oxide substrate around room temperature,^21^ but by 495 K, they diffuse out of that interface, most likely (according to the XANES data in Figure 3C) to the subsurface region within the Cu NPs. Such transformation is reversible, since the Pt returns to the NP/silica interface once the sample is cooled back down to 295 K (Figures 2 and 3). This temperature-driven interconversion is aided by exposure to an H_2_ atmosphere, as it does not happen under a pure He environment (Figure S4b). While we do not have a full explanation for this phenomenon, we speculate that it may be associated with changes in the relative energetics for molecular vs atomic hydrogen adsorption as a function of the position of the Pt atom within the alloy NPs. As shown in Figures S7 and S8, the difference between those two energies is the largest with Pt inside the NPs, a fact that shifts the equilibrium between atomic hydrogen adsorption on clusters with the Pt at the interface vs inside the metal NP toward the latter as the temperature is increased. There may also be entropic factors at play here. This issue requires further investigation.

It should also be noted that although the Pt atoms in the initial CuPt_0.005_/SBA-15 appear to be fully dispersed in atomic form within the metal NPs (Figures 1, S1, and S2), the EXAFS results reported in Figures 2 and S4 and in Table S1 indicate that they form small aggregates once exposed to a reactive environment. Indeed, the average coordination number for Pt atoms bonded to other Pt atoms under an H_2_ atmosphere was estimated to be approximately 2 at room temperature, suggesting that they may form small (∼3 atoms) clusters at the NP/silica interface. The Pt–Pt coordination number is lower when heating the catalyst to 495 K (in H_2_), meaning that as the Pt atoms diffuse to the subsurface or surface of the metal NPs they may detach from other Pt atoms. It is difficult to extract detailed structural information on these Pt aggregates from the XAS data, but the XANES spectra could be best fitted to structures consistent with some Pt still binding to the NP/silica interface at 295 K but with other Pt atoms inside the cluster at 495 K (Figure S9). DFT calculations (performed with larger, Cu_82_Pt_2_/SiO_2_(111), clusters) are also inconclusive on this point, but they also suggest that the additional Pt atoms may prefer positions within the inside of the alloy NPs (Figure S10). This may in fact be the reason why the phenomenon reported here had not been detected before: possibly, the dopant atoms that end up in the bulk of the alloy NPs at higher dopant contents may mask the spectroscopic signals associated with the initial interfacial dopant atoms. In any case, the changes seen during temperature cycling of the catalyst are reversible, and the most likely scenario during active hydrogenation catalysis is still one where the Pt atoms are in the form of small clusters at the NP/silica interface. Calling these catalysts single-atom alloys (SAAs) may therefore be a misnomer that does not accurately represent their nature under reaction conditions. We also believe that such dopant atom clustering at the NP/support interface may happen with other so-called SAA systems, only that the effect may be masked and difficult to detect unless very diluted (<1 atom %) alloys and *in situ* conditions are used in the characterization studies (as we have done here).

Finally, temperature cycling of these dilute alloy catalysts under CO atmospheres may also help with the Pt segregation process although the EXAFS data in Figure S4b suggest that CO is not as effective as H_2_ in promoting such a transformation. Additional evidence for CO-induced segregation to the NP surface at high temperatures was acquired by infrared absorption spectroscopy (Figure S11). Metal diffusion in and out of the surface upon exposure to reactive gases is a well-known phenomenon and has been reported for many alloys,^11,26^ including Cu–Pt SAAs in both bulk single crystals^12^ and with supported NPs.^21^ What is unique about our results is the fact that the preferred and most stable configuration of Pt in Cu–Pt diluted alloys is at the inner NP/silica interface and that such Pt can still remotely promote the dissociation of adsorbed molecular hydrogen several layers away. We are not aware of such behavior being reported in the past. The remote H_2_ activation is likely to be a key step in hydrogenation catalysis. This behavior is also likely to extend to other SAAs.

## 4 Conclusions

The behavior of dilute alloys comprised of Cu NPs containing small amounts of Pt and dispersed on a silica (SBA-15) support was characterized *in situ* in the presence of reactive (H_2_, CO) atmospheres. They were found to behave differently than when exposed to adsorbates under vacuum and to exhibit a dynamic and reversible behavior driven by temperature and the nature of the gas. First, they show different activity and selectivity than the pure metals, reaching optimum performance for the hydrogenation of unsaturated aldehydes to unsaturated alcohols with Pt contents around 0.5 atom %. This was found to be the case even though it was determined, mainly on the basis of results from *in situ* XAS experiments, that the Pt atoms prefer to diffuse into the inside interface between the NPs and the silica support when exposed to H_2_ at room temperature. DFT calculations not only confirmed the location of the Pt atoms at such interface because of the additional stability provided by the formation of Pt–Si bonds but also indicated that the Pt atoms can still modify the electronic density of the Cu atoms located at the surface, two to three lattice distances away. The electronic changes induced by the subsurface Pt lead to a lowering of both the adsorption energy of H_2_ and the activation energy for its dissociation, thus facilitating the uptake of the hydrogen atoms on the surface needed for the hydrogenation of unsaturated organic reactants.

A few additional observations derived from our experiments are worth keeping in mind when considering the reaction mechanisms of catalytic processes. For one, the Pt atoms were found to diffuse back to the surface (or the near surface) upon heating of the catalyst (in a hydrogen atmosphere). This segregation is reversible, and the Pt atoms return to the NP/silica interface once the catalyst is cooled back to room temperature. A similar behavior was seen, albeit less pronounced, with CO (as also corroborated by IR experiments), but not with inert gases such as He. Finally, even though the catalyst characterized here contains a very dilute (0.5 atom %) amount of Pt, those atoms were still found to pair up to some degree. It may be that under reaction conditions, the so-called single-atom alloy (SAA) catalysts may not really be SAAs. An additional open question concerns the placement of the new atoms as the alloys are enriched in Pt. Our initial calculations appear to indicate that those eventually end up in the NP bulk rather than at the interface, a possible reason why the interfacial Pt atoms have not been reported in previous SAA studies.^21^

## Supporting Information Available

CuPt_0.005_/SBA-15 TEM-EDX line scans and HRTEM images; EXAFS data analysis and fitting parameters; Cu_24_Pt cluster energy stabilization by SiO_2_; Cu–Pt SAA DOS, bulk vs NPs; energetics and structures of H_2_ vs 2H adsorption on Cu_24_Pt/SiO_2_(111); simulated XANES for Cu_23_Pt_2_/SiO_2_; DFT calculations of Pt_2_-containing CuPt_*x*_/SiO_2_(111) clusters; IR data for CO adsorption on CuPt_*x*_/SBA-15 (PDF).
