# Supplementary material for: Remote Activation of H–H Bonds by Platinum in Dilute Alloy Catalysts
Source: ACS Catal. 2024 Apr 23;14(9):7157–65. doi: 10.1021/acscatal.4c00886 (PMC11075014; doi:10.1021/acscatal.4c00886)
Supplement: Supplementary file 1 — cs4c00886_si_001.pdf [file cs4c00886_si_001.pdf]

Remote Activation of H–H Bonds by Platinum in Single-Atom Alloy Catalysts

Tongxin Han,[1] Yuanyuan Li,[2,&] Tao Wu,[3] Debora Motta Meira,[4,5] Shuting Xiang,[2] Yueqiang Cao,[1,6] Ilkeun Lee,[1] Xinggui Zhou,[6] De-en Jiang,[7] Anatoly I. Frenkel,[2,8] and Francisco Zaera[1,*]

[1] Department of Chemistry and UCR Center for Catalysis, University of California, Riverside, CA 92521, USA.

[2] Department of Materials Science and Chemical Engineering, Stony Brook University, Stony Brook, NY 11794, USA.

[3] The State Key Laboratory of Fine Chemicals, School of Chemical Engineering, Dalian University of Technology, Dalian 116024, P. R. China.

[4] CLS@APS, Advanced Photon Source, Argonne National Laboratory, Argonne, IL 60439, USA.

[5] Canadian Light Source Inc., 44 Innovation Boulevard, Saskatoon, Saskatchewan S7N 2V3, Canada.

[6] State Key Laboratory of Chemical Engineering, School of Chemical Engineering, East China University of Science and Technology, Shanghai 200237, P. R. China.

[7] Department of Chemical and Biomolecular Engineering, Vanderbilt University, Nashville, TN 37212, USA.

[8] Chemistry Division, Brookhaven National Laboratory, Upton, NY 11973, USA.

[&] Present address: Chemical Sciences Division, Oak Ridge National Laboratory, Oak Ridge, TN 37831, USA.

* Corresponding Author, Email: zaera@ucr.edu

Supplementary Information

1. Fig. S1. $CuPt_{0.005}$/SBA-15 TEM-EDX Line Scans. 3

2. Fig. S2. $CuPt_{0.005}$/SBA-15 HRTEM Images. 4

3. Fig. S3. $CuPt_{0.005}$/SBA-15 Cu K-Edge XAS Data Before & After Reduction. 5

4. Fig. S4a. EXAFS Data Analysis: Details of Data Fitting. 6

5. Fig. S4b. EXAFS Data Analysis: Data Fitting versus Gas and Temperature. 7

6. Table S1. EXAFS Fitting Parameters. 8

7. Fig. S5. $Cu_{24}Pt$ Cluster Energy Stabilization by SiO_2. 9

8. Fig. S6. Cu-Pt SAA DOS, Bulk vs. NPs. 10

9. Fig. S7. Energetics of H_2 vs. 2H Adsorption on $Cu_{24}Pt/SiO_2(111)$. 11

10. Fig. S8. Structures for H_2 vs. 2H Adsorption on $Cu_{24}Pt/SiO_2(111)$. 12

11. Fig. S9. Simulated XANES for $Cu_{23}Pt_2/SiO_2$. 13

12. Fig. S10. DFT Calculations of Pt_2-Containing $CuPt_x/SiO_2(111)$ Clusters. 15

13. Fig. S11. IR Data for CO Adsorption on $CuPt_x$/SBA-15. 16

1. Fig. S1. $CuPt_{0.005}$/SBA-15 TEM-EDX Line Scans.

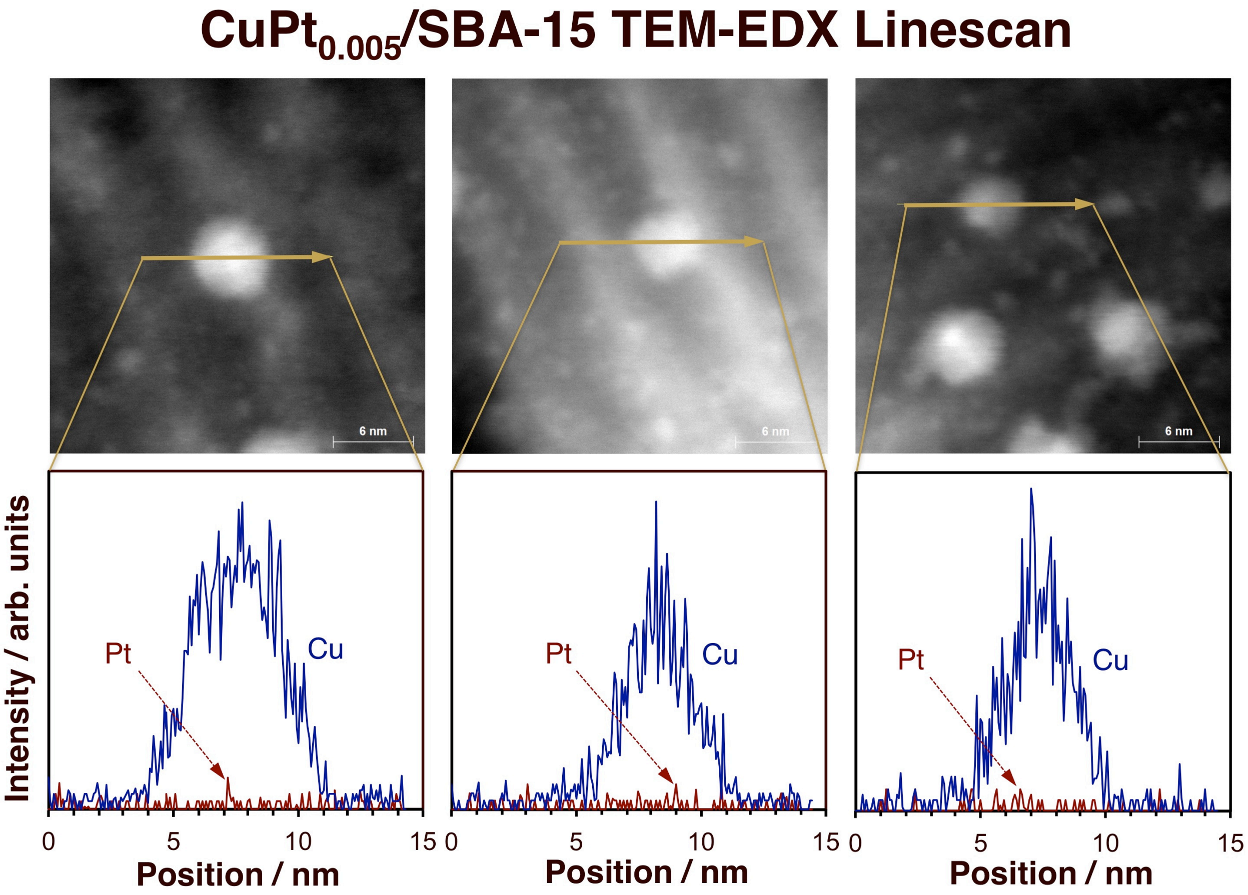

Evidence of the atomic dispersion of the Pt atoms is indicated by the occasional signal blips in the EDX scans for Pt (red traces).

2. Fig. S2. $CuPt_{0.005}$/SBA-15 HRTEM Images.

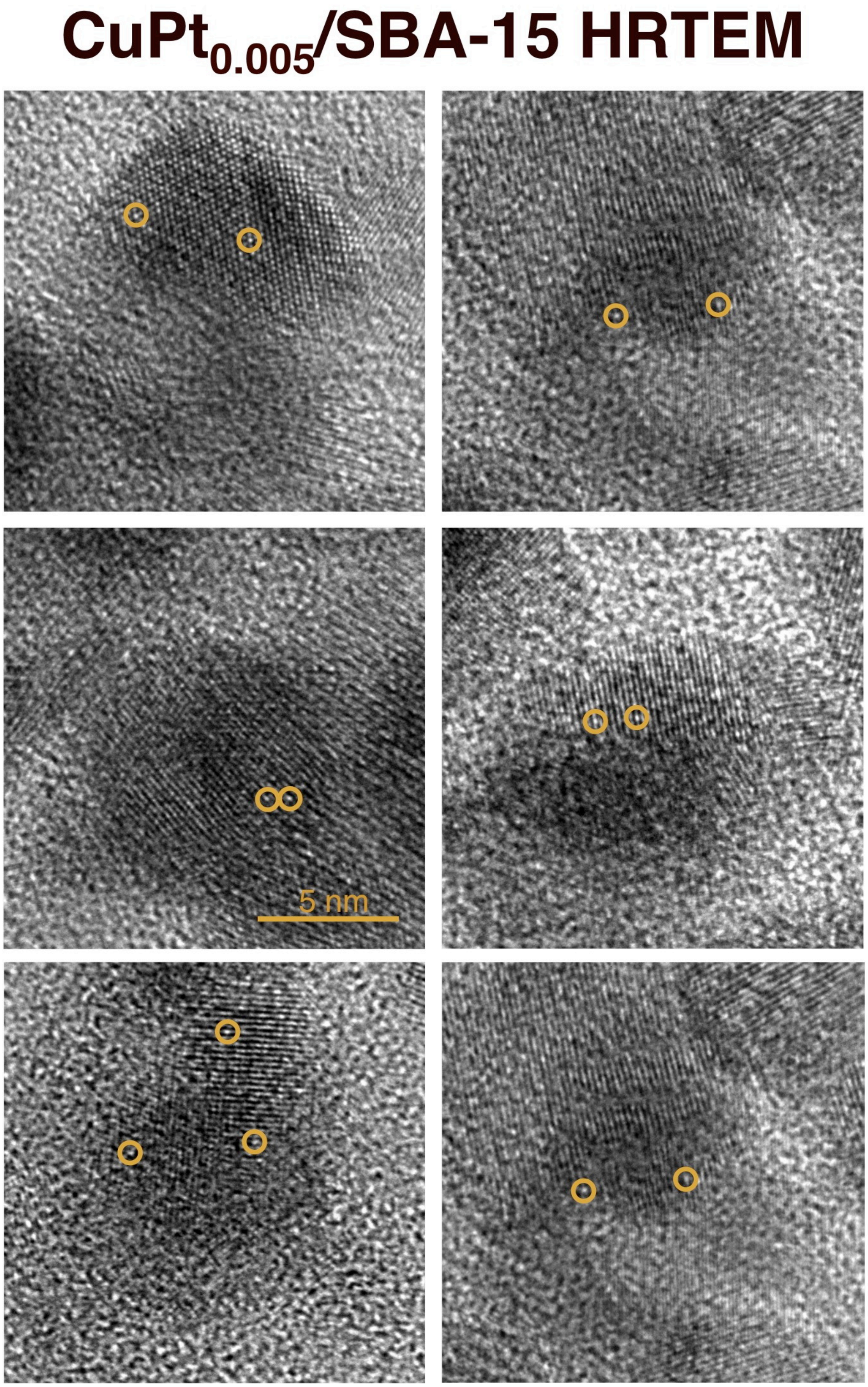

Evidence of the atomic dispersion of the Pt atoms (some of which are highlighted by yellow circles) within the Cu-Pt alloy NPs in the initial $CuPt_{0.005}$/SBA-15 catalyst.

3. **Fig. S3. $CuPt_{0.005}$/SBA-15 Cu K-Edge XAS Data Before & After Reduction.**

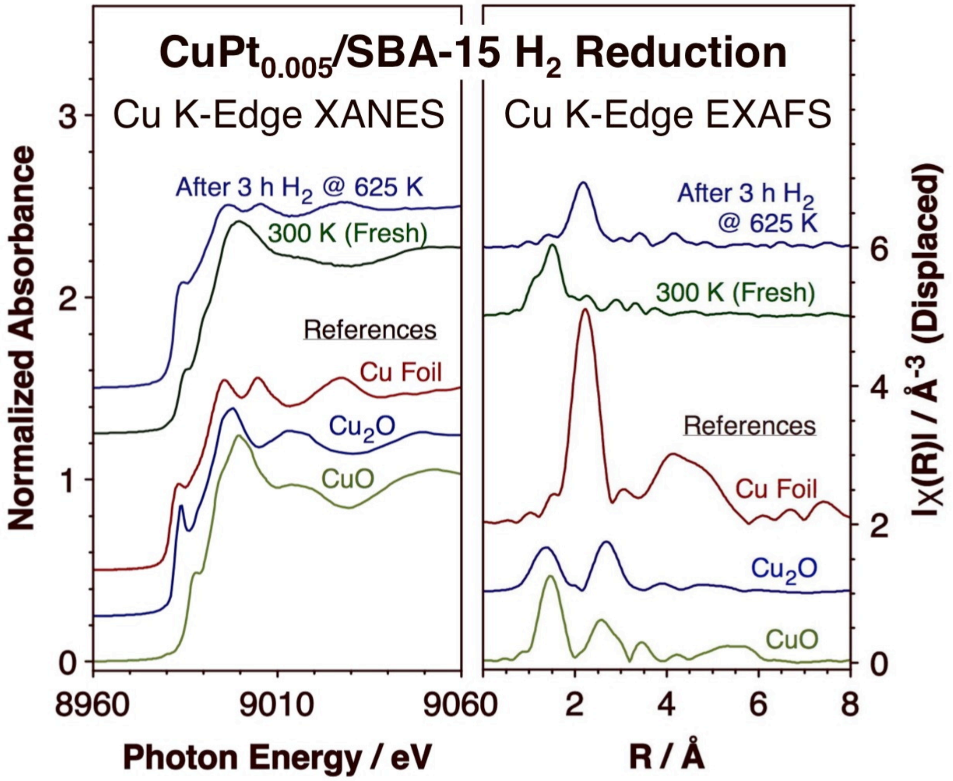

Cu K-edge XANES (left panel) and EXAFS radial distribution (right) data for our $CuPt_{0.005}$/SBA-15 catalyst as prepared (300 K – Fresh, green traces) and after reduction for 3 h in H_2 at 625 K (blue traces). Additional data taken for a Cu foil and for Cu_2O and CuO samples are included for reference. The data indicate that the Cu in our catalyst is initially in an oxidized (CuO) form but is fully reduced to its metallic state after the H_2 pretreatment.

4. Fig. S4a. EXAFS Data Analysis: Details of Data Fitting.

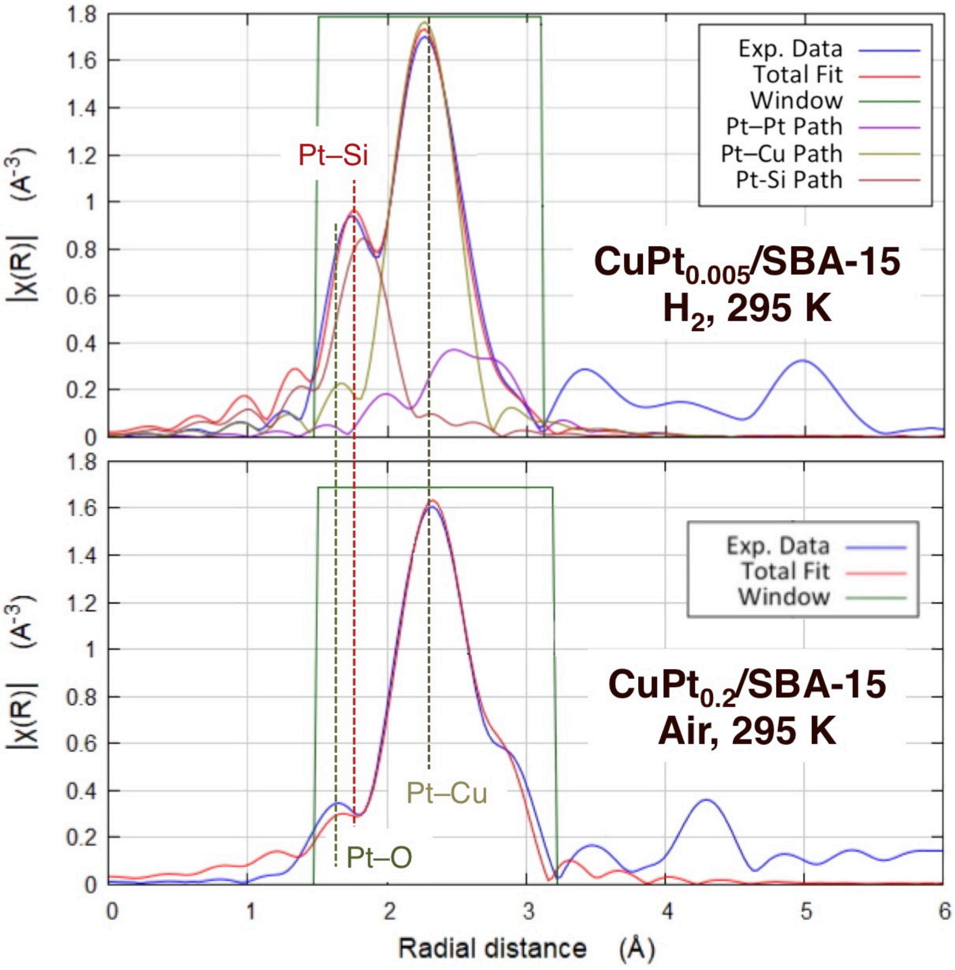

Top: Details of the fit of *in situ* Pt L_3-edge EXAFS data for $CuPt_{0.005}$/SBA-15 obtained under 1 bar of 5 vol% H_2 in N_2 at 295 K, the first of the set of experiments reported in Figure S4b. All individual scattering paths used for the fit are shown to highlight the need to add the Pt–Si path to account for the low radial-distance peak. Bottom: Similar *in situ* Pt L_3-edge EXAFS data and analysis for a $CuPt_{0.2}$/SBA-15 catalyst exposed to air at 295 K, provided to illustrate the approximately 0.1 Å shift in peak position expected for a Pt–O bond (seen in this sample) versus a Pt–Si bond (seen with $CuPt_{0.005}$/SBA-15).

5. Fig. S4b. EXAFS Data Analysis: Data Fitting versus Gas and Temperature.

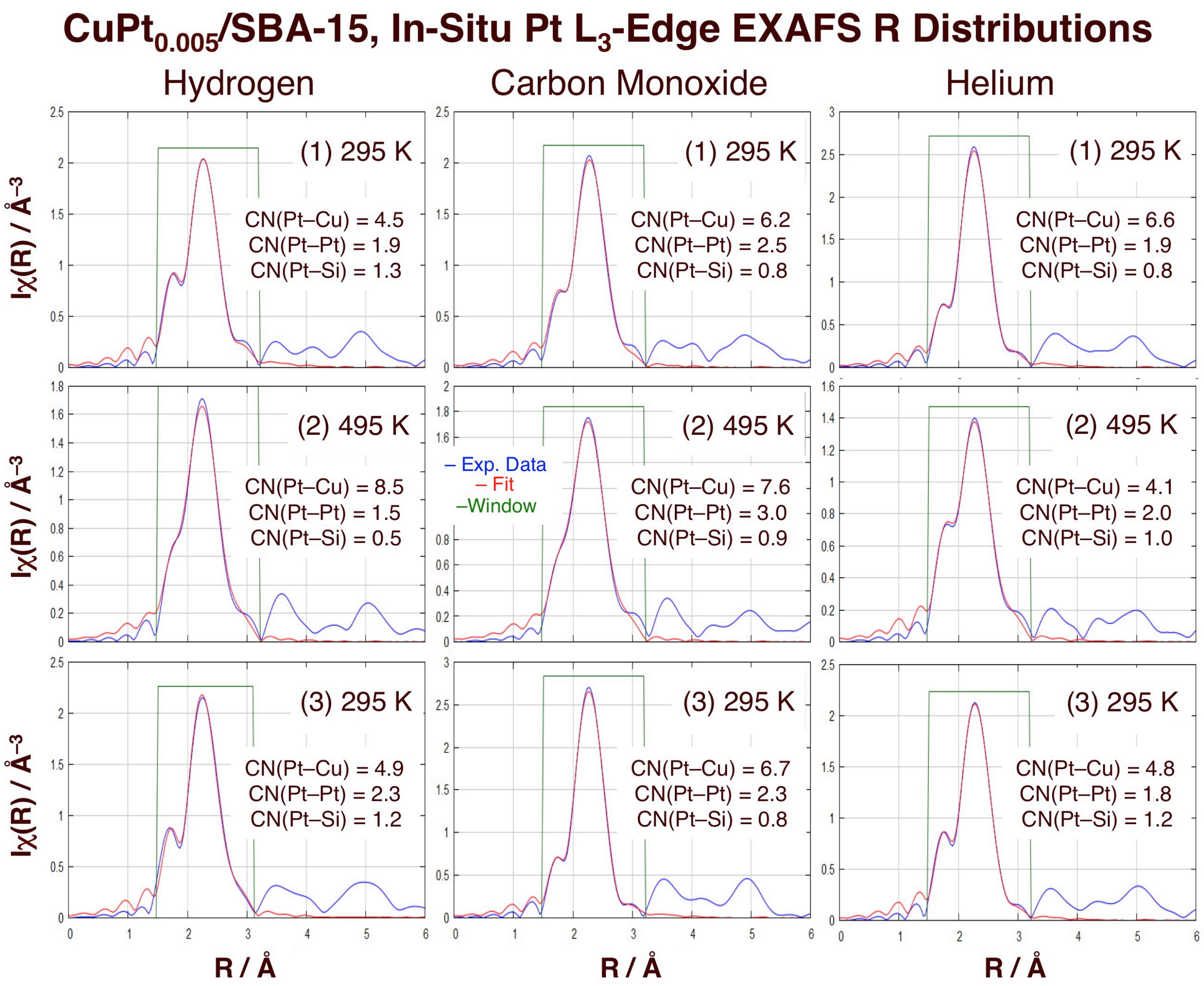

Fit of *in situ* Pt L_3-edge EXAFS data for $CuPt_{0.005}$/SBA-15 under 1 bar of 5 vol% H_2 in N_2 (left column), 5 vol% CO in He (center), and pure He (right) as the temperature was cycled from 295 K (top row) to 495 K (center) and back to 295 K (bottom). The red traces correspond to the curves obtained by fitting the experimental data (blue traces) inside the green windows. The resulting coordination numbers (CNs) for Pt–Cu, Pt–Pt, and Pt–Si are provided inside each panel.

6. Table S1. EXAFS Fitting Parameters.

Gas[a]	T / K	CN(Pt–Si)	CN(Pt–Cu)	CN(Pt–Pt)	CN(Total)	R(Pt–Si)/Å	R(Pt-Cu)/Å	R(Pt–Pt)/Å	σ^2(Pt–Cu)/Å^2	σ^2(Pt–Pt)/Å^2	ΔE_0/eV	R Factor
5% H_2	295	1.4 ± 0.3	4.1 ± 1.4	2.0 ± 0.6	7.5 ± 1.6	2.24 ± 0.02	2.58 ± 0.01	2.72 ± 0.03	0.004 ± 0.003	0.004 ± 0.003	6 ± 3	0.004
5% H_2	495	0.4 ± 0.5	10.6 ± 3.4	0	11.0 ± 3.4	2.27 ± 0.06	2.58 ± 0.02		0.011 ± 0.003		6 ± 3	0.01
5% H_2	295	1.3 ± 0.3	4.8 ± 1.2	1.8 ± 0.5	7.9 ± 1.3	2.26 ± 0.02	2.59 ± 0.01	2.75 ± 0.02	0.003 ± 0.002	0.003 ± 0.002	8 ± 2	0.003
5% H_2	495	0.7 ± 0.4	6.5 ± 2.2	2.9 ± 1.1	10.1 ± 2.5	2.26 ± 0.04	2.58 ± 0.02	2.75 ± 0.04	0.007 ± 0.003	0.007 ± 0.003	8 ± 3	0.006
5% H_2	295	1.1 ± 0.3	5.1 ± 1.4	2.8 ± 0.7	9.0 ± 1.6	2.23 ± 0.03	2.57 ± 0.01	2.72 ± 0.02	0.004 ± 0.002	0.004 ± 0.002	6 ± 2	0.003
5% CO	295	0.9 ± 0.3	5.3 ± 1.3	2.2 ± 0.6	8.4 ± 1.5	2.26 ± 0.03	2.59 ± 0.01	2.74 ± 0.03	0.004 ± 0.002	0.004 ± 0.002	8 ± 2	0.003
5% CO	495	0.8 ± 0.4	6.5 ± 2.1	3.0 ± 1.1	10.3 ± 2.4	2.27 ± 0.04	2.59 ± 0.02	2.76 ± 0.04	0.007 ± 0.002	0.007 ± 0.002	9 ± 3	0.005
5% CO	295	0.7 ± 0.3	7.1 ± 1.5	1.7 ± 0.7	9.5 ± 1.7	2.26 ± 0.03	2.59 ± 0.01	2.74 ± 0.03	0.004 ± 0.001	0.004 ± 0.001	7 ± 2	0.003
He	295	0.8 ± 0.3	6.6 ± 1.5	1.9 ± 0.7	9.3 ± 1.7	2.26 ± 0.03	2.59 ± 0.01	2.74 ± 0.03	0.004 ± 0.002	0.004 ± 0.002	8 ± 2	0.003
He	495	1.0 ± 0.3	4.1 ± 1.3	2.0 ± 0.6	7.1 ± 1.5	2.27 ± 0.03	2.60 ± 0.01	2.76 ± 0.03	0.005 ± 0.002	0.005 ± 0.002	9 ± 3	0.005
He	395	1.2 ± 0.3	4.8 ± 1.2	1.8 ± 0.5	7.8 ± 1.3	2.26 ± 0.02	2.59 ± 0.01	2.73 ± 0.03	0.003 ± 0.002	0.003 ± 0.002	8 ± 2	0.003
5% CO	295	0.8 ± 0.3	6.3 ± 1.4	2.3 ± 0.6	9.4 ± 1.5	2.26 ± 0.03	2.59 ± 0.01	2.74 ± 0.03	0.005 ± 0.002	0.005 ± 0.002	7 ± 2	0.003
5% CO	495	1.0 ± 0.5	7.6 ± 2.9	0	8.6 ± 2.9	2.28 ± 0.04	2.60 ± 0.02		0.008 ± 0.003		9 ± 4	0.01
5% CO	295	0.7 ± 0.3	6.9 ± 1.4	3.0 ± 0.6	10.6 ± 1.6	2.27 ± 0.03	2.59 ± 0.01	2.75 ± 0.02	0.005 ± 0.001	0.005 ± 0.001	8 ± 2	0.002
5% CO	495	0.8 ± 0.5	8.7 ± 3.0	0	9.5 ± 3	2.28 ± 0.04	2.60 ± 0.02		0.009 ± 0.003		8 ± 3	0.01
5% CO	295	0.9 ± 0.3	6.2 ± 1.6	2.2 ± 0.6	9.3 ± 1.7	2.26 ± 0.04	2.60 ± 0.01	2.75 ± 0.03	0.003 ± 0.002	0.003 ± 0.02	6 ± 3	0.004

[a] H_2 was diluted in N_2, 5 vol%, CO was diluted in He, 5 vol%. Total Flow = 10 mL/min in all cases.

Geometrical parameters for the coordination around the Pt atoms in the $CuPt_{0.005}$/SBA-15 catalyst under different gas atmospheres. Data extracted from fits of the *in situ* EXAFS data. Note: The values for σ^2(Pt–Si) came out to be below the experimental limit (three significant figures after the decimal point) in all cases, meaning that the corresponding coordination numbers, CN(Pt–Si), are lower bounds of their actual values.

7. Fig. S5. $Cu_{24}Pt$ Cluster Energy Stabilization by SiO_2.

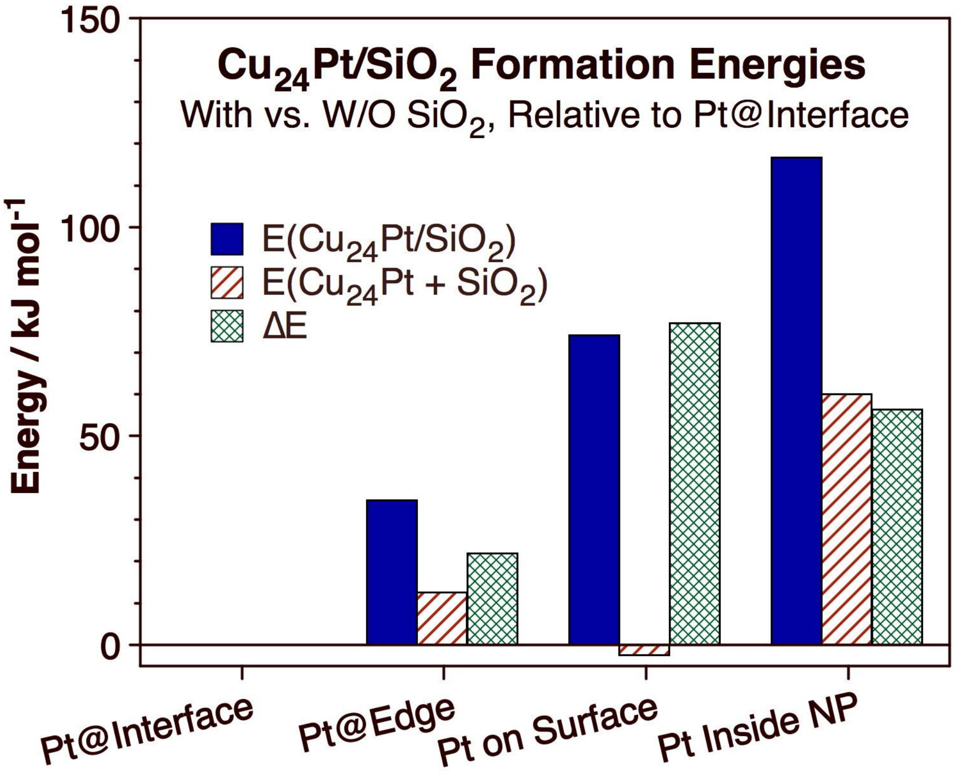

DFT calculations of the relative energies required to create $Cu_{24}Pt$ clusters by themselves and supported on a $SiO_2(111)$ surface as a function of the position of the Pt atom within the metal cluster. All energies are estimated relative to the Pt@Interface case, used as reference. The supported cluster is the most stable when the Pt atom sits at the interface in great part because of the energy provided by the Pt–Si bond associated with that configuration. This fact is highlighted in the figure by the trend seen in the values estimated for $\Delta E = E(C_{24}Pt/SiO_2) - E(Cu_{24}Pt + SiO_2)$ (green crosshatched bars).

8. **Fig. S6. Cu-Pt SAA DOS, Bulk vs. NPs.**

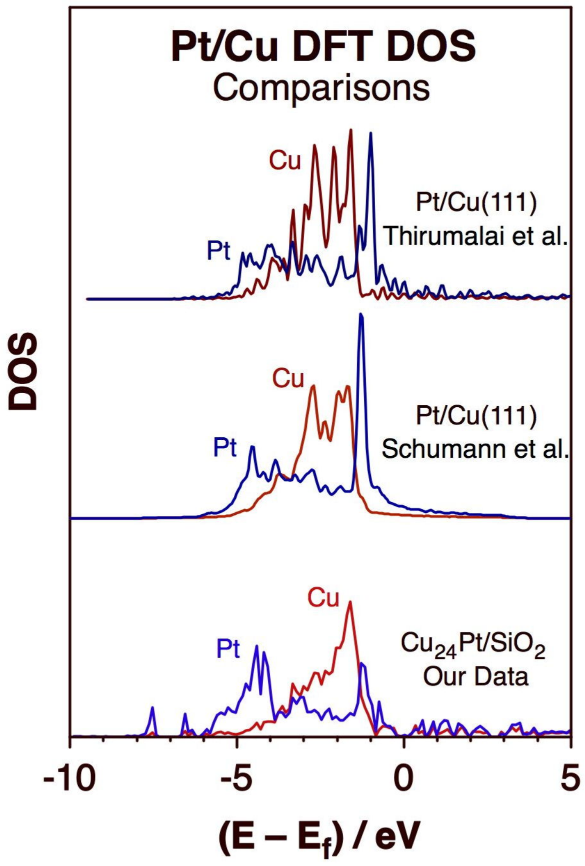

Comparison of the Pt (blue) and Cu (red) DFT-calculated DOS of $Cu_{24}Pt/SiO_2(111)$ SAAs NP catalysts (bottom, our calculations) versus in Pt/Cu(111) single crystals, from refs. (*12*) (center) and (*13*) (top). In the NP case, some of the DOS of Pt is transferred from ~-1.2 eV to the –4 to –5 eV region, and there is less Cu DOS in the –2 to –3 eV region.

9. **Fig. S7. Energetics of H_2 vs. 2H Adsorption on $Cu_{24}Pt/SiO_2(111)$.**

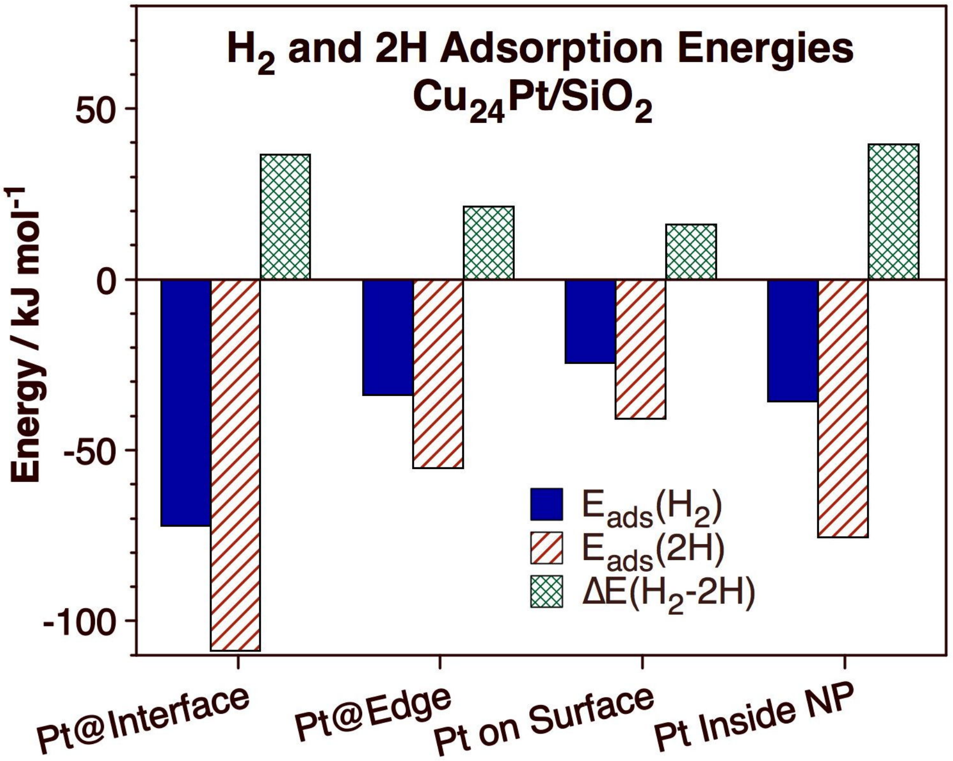

Energies calculated for the adsorption of H_2 and 2H on $Cu_{24}Pt/SiO_2(111)$ catalysts as a function of the position of the Pt atom within the cluster. The difference between the adsorption of a H_2 molecule and two H atoms is the largest with the Pt atom inside the $Cu_{24}Pt$ cluster, a fact that brings the energetics of 2H adsorption in that case closer to the value for Pt@Interface. This can help shift the equilibria toward the former system at higher temperatures.

10. **Fig. S8. Structures for H_2 vs. 2H Adsorption on $Cu_{24}Pt/SiO_2(111)$.**

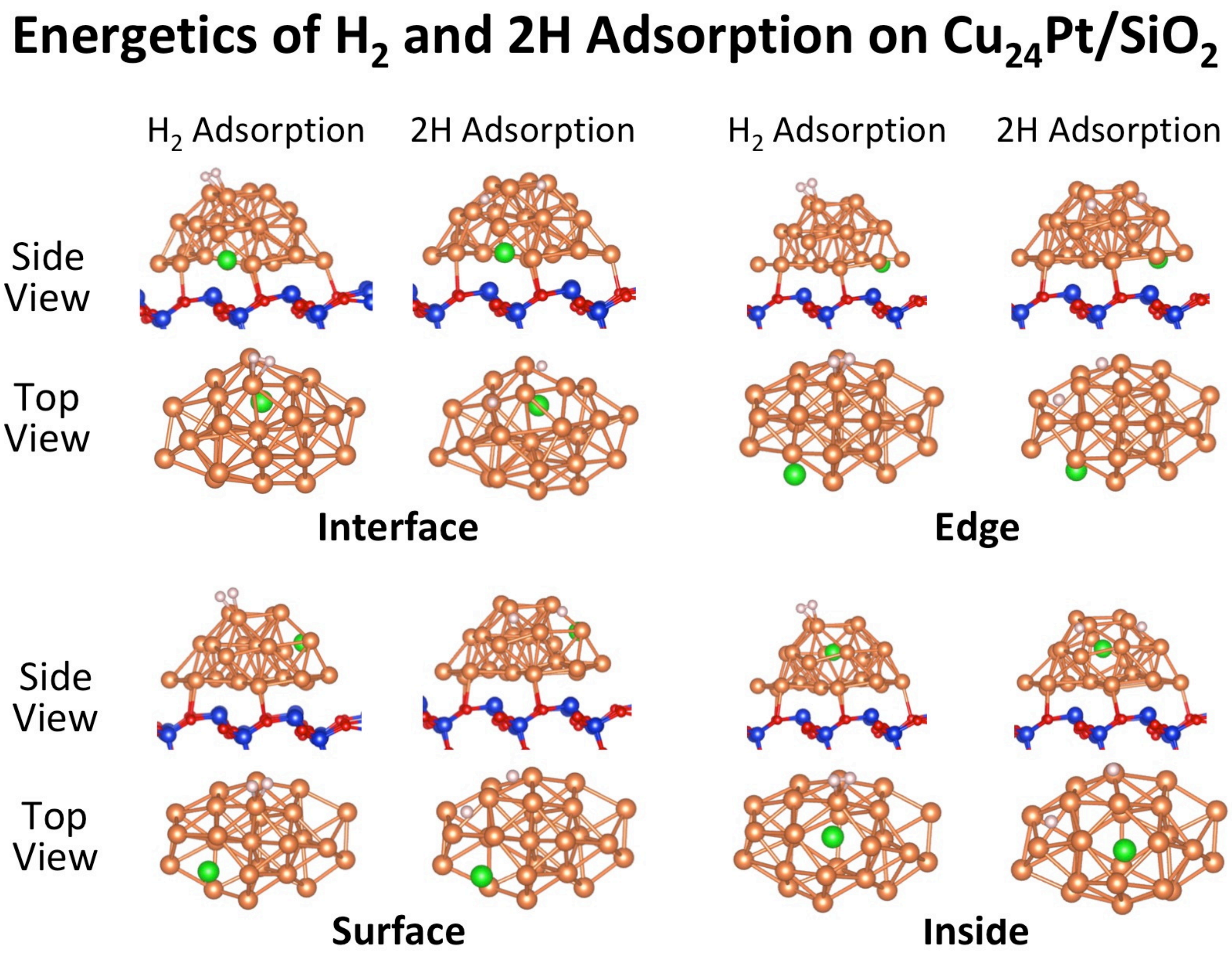

These are the optimized structures for the cases reported in Figure S7.

11. Fig. S9. Simulated XANES for $Cu_{23}Pt_2/SiO_2$.

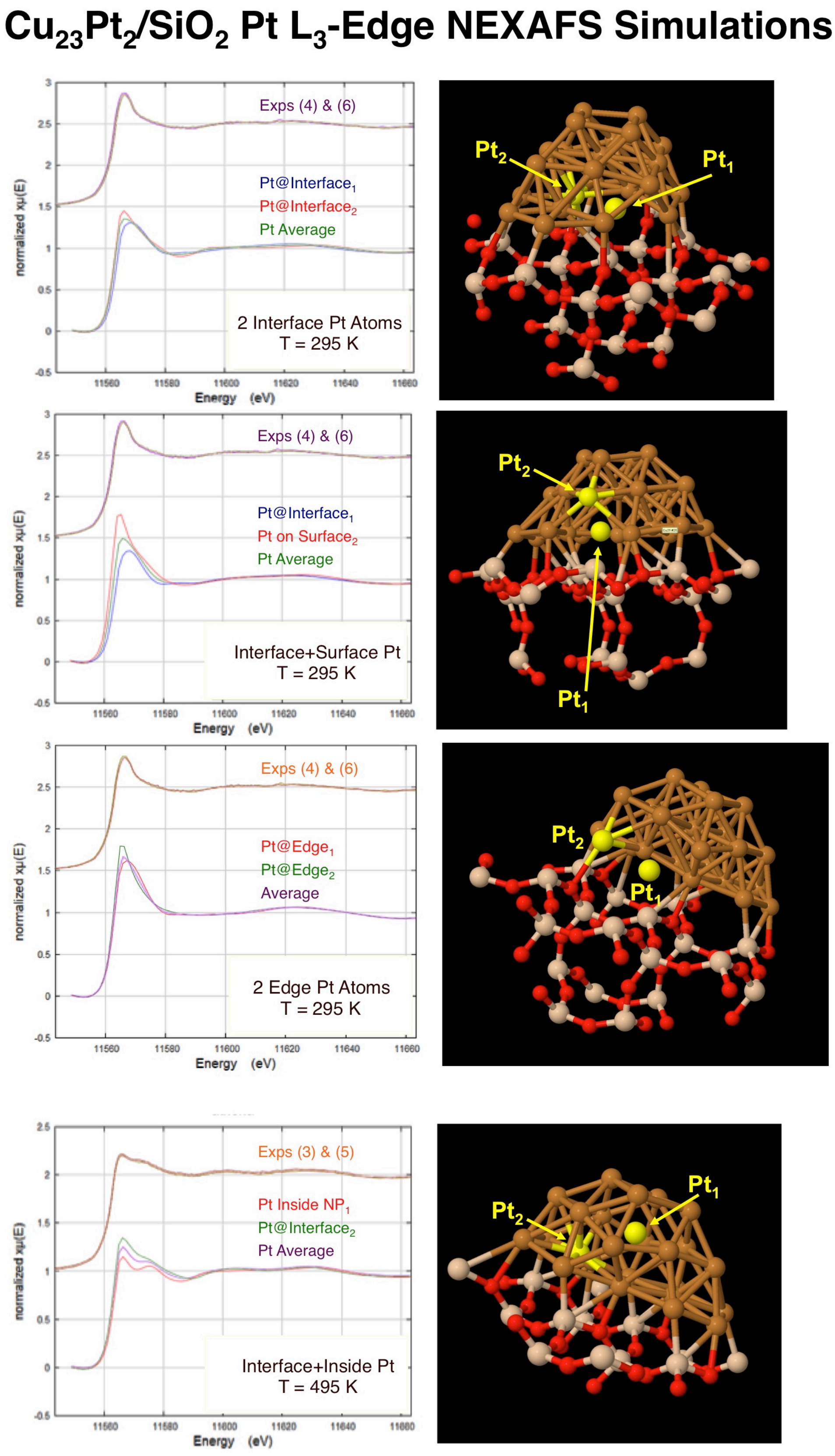

Best simulated XANES spectra consistent with the experimental data, used to estimate the possible locations of the Pt atoms within the Pt-Cu alloy clusters. In each case, three simulated spectra are reported, for each of the two individual Pt atoms (Pt_1 and Pt_2), and for the average of the two. The structures of the clusters used for the simulations and the locations of the Pt atoms within them are provided in the right column.

Top: Comparison of the experimental XANES data for 295 K (experiments 4 and 6) with simulated spectra for the two Pt atoms located at the NP/silica interface.

Second from Top: Comparison of the experimental XANES data for 295 K (experiments 4 and 6) with simulated spectra for one Pt atom at the NP/silica interface and the second at the adjacent NP surface site.

Third from Top: Comparison of the experimental XANES data for 295 K (experiments 4 and 6) with simulated spectra for the two Pt atoms at the edge of the NP/silica interface.

The simulations in all these three cases reproduce most of the features of the experimental XANES data from $CuPt_{0.005}$/SBA-15 exposed to a H_2 atmosphere at 295 K.

Bottom: Comparison of the experimental XANES data for 495 K (experiments 3 and 5) with simulated spectra for one Pt atoms located in the inside of the metal NP and the other at an inner NP/silica interface site. This is the case that provided the best match with the experimental XANES data from $CuPt_{0.005}$/SBA-15 exposed to a H_2 atmosphere at 495 K

12. Fig. S10. DFT Calculations of Pt_2-Containing $CuPt_x/SiO_2(111)$ Clusters.

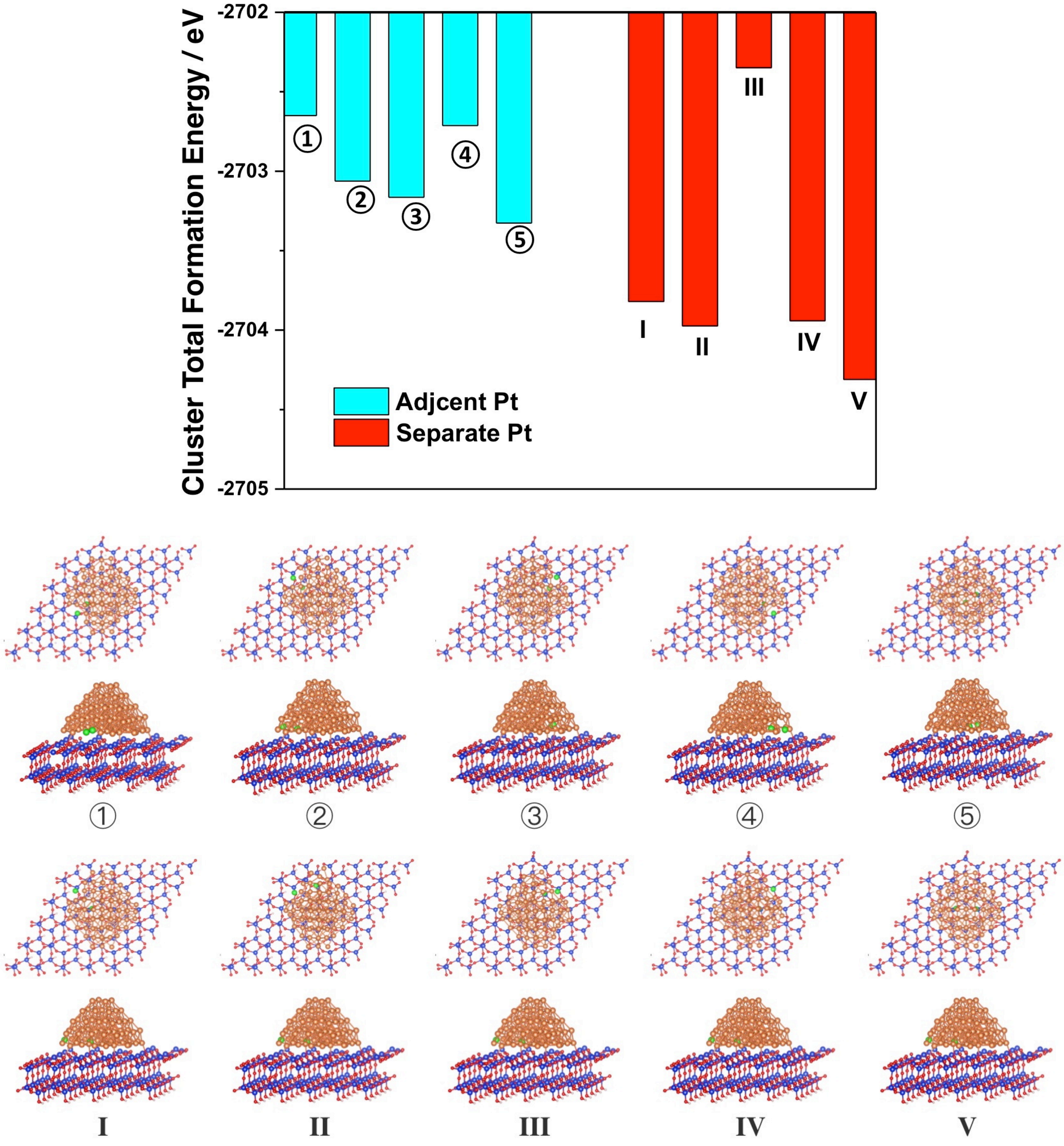

Comparison of energetics of $Cu_{82}Pt_2/SiO_2(111)$ clusters, with the Pt atoms in adjacent (numbers in circles, blue bars, top row) versus separate (roman numerals, red bars, bottom row) relative positions.

13. Fig. S11. IR Data for CO Adsorption on $CuPt_x$/SBA-15.

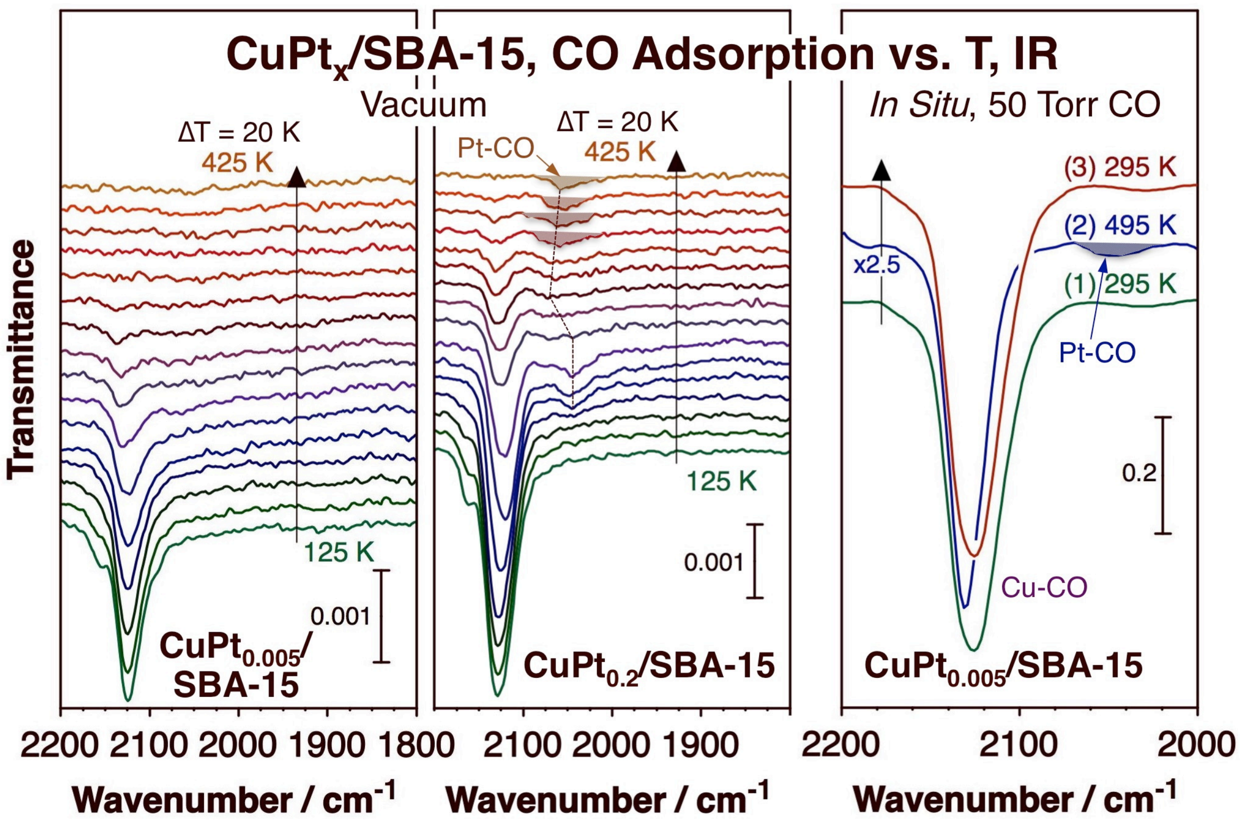

IR absorption spectra for CO adsorbed on $CuPt_{0.005}$/SBA-15 (left panel) and $CuPt_{0.2}$/SBA-15 (center) catalysts under vacuum as a function of temperature. The main peak at 2123 cm^{-1} is due to adsorption on Cu; desorption from that surface is complete after heating to room temperature. CO adsorption on Pt, which is detected at lower frequencies (below 2100 cm^{-1}), cannot be seen for the diluted alloy ($CuPt_{0.005}$/SBA-15; left panel) catalyst, but is visible in the spectra for $CuPt_{0.2}$/SBA-15 (center panel). In the presence of CO gas, on the other hand, CO adsorption is detectable even on $CuPt_{0.005}$/SBA-15*,* but mainly at 495 K (right panel). The new IR feature is barely detectable initially and goes away again upon cooling down from 495 to 295 K, indicating the reversibility of the Pt atom segregation.
